# Smartphone-Based Remote Monitoring for Chronic Heart Failure: Mixed Methods Analysis of User Experience From Patient and Nurse Perspectives

**DOI:** 10.2196/44630

**Published:** 2023-06-06

**Authors:** Alice Auton, Sameer Zaman, Yorissa Padayachee, Jack W Samways, Nicholas M Quaife, Mark Sweeney, Indira Tenorio, Nick W F Linton, Graham D Cole, Nicholas S Peters, Jamil Mayet, Carys Barton, Carla Plymen

**Affiliations:** 1 Imperial College Healthcare NHS Trust London United Kingdom; 2 Imperial College London London United Kingdom

**Keywords:** heart failure, health-related quality of life, mHealth, nurse specialist, patient engagement, self-management, self-care

## Abstract

**Background:**

Community-based management by heart failure specialist nurses (HFSNs) is key to improving self-care in heart failure with reduced ejection fraction. Remote monitoring (RM) can aid nurse-led management, but in the literature, user feedback evaluation is skewed in favor of the patient rather than nursing user experience. Furthermore, the ways in which different groups use the same RM platform at the same time are rarely directly compared in the literature. We present a balanced semantic analysis of user feedback from patient and nurse perspectives of Luscii, a smartphone-based RM strategy combining self-measurement of vital signs, instant messaging, and e-learning.

**Objective:**

This study aims to (1) evaluate how patients and nurses use this type of RM (usage type), (2) evaluate patients’ and nurses’ user feedback on this type of RM (user experience), and (3) directly compare the usage type and user experience of patients and nurses using the same type of RM platform at the same time.

**Methods:**

We performed a retrospective usage type and user experience evaluation of the RM platform from the perspective of both patients with heart failure with reduced ejection fraction and the HFSNs using the platform to manage them. We conducted semantic analysis of written patient feedback provided via the platform and a focus group of 6 HFSNs. Additionally, as an indirect measure of tablet adherence, self-measured vital signs (blood pressure, heart rate, and body mass) were extracted from the RM platform at onboarding and 3 months later. Paired 2-tailed *t* tests were used to evaluate differences between mean scores across the 2 timepoints.

**Results:**

A total of 79 patients (mean age 62 years; 35%, 28/79 female) were included. Semantic analysis of usage type revealed extensive, bidirectional information exchange between patients and HFSNs using the platform. Semantic analysis of user experience demonstrates a range of positive and negative perspectives. Positive impacts included increased patient engagement, convenience for both user groups, and continuity of care. Negative impacts included information overload for patients and increased workload for nurses. After the patients used the platform for 3 months, they showed significant reductions in heart rate (*P*=.004) and blood pressure (*P*=.008) but not body mass (*P=*.97) compared with onboarding.

**Conclusions:**

Smartphone-based RM with messaging and e-learning facilitates bilateral information sharing between patients and nurses on a range of topics. Patient and nurse user experience is largely positive and symmetrical, but there are possible negative impacts on patient attention and nurse workload. We recommend RM providers involve patient and nurse users in platform development, including recognition of RM usage in nursing job plans.

## Introduction

Despite effective treatment options, patients with chronic heart failure with reduced ejection fraction (HFrEF) have a low quality of life [1]. The importance of improving patient self-management is appreciated by international clinical guidelines including the European Society of Cardiology [2], but achieving this remains a major challenge in HFrEF care [3]. Regular clinical review by heart failure specialist nurses (HFSNs) is the cornerstone of community-based management. This includes a range of tasks such as monitoring patient-measured vital signs, asking about symptoms, uptitrating prognostic medication doses, altering diuretic doses, answering ad hoc questions, and delivering education for patients and carers. Although HFSN management decreases hospital admissions [4] and nurse-led education is known to improve quality of life [5], there is no consensus on the optimal way to deliver this care.

Community management by HFSNs typically relies on high-frequency monitoring of vital signs and regular symptom review via serial face-to-face outpatient appointments [6]. In practice, not only are these appointments burdensome for patients to attend, but there is no systematic way to capture rapid changes in patients’ clinical states between the appointments, so that timely intervention can be provided. This potentially misses a window of opportunity, which may lead to increased morbidity and worse quality of life.

Remote monitoring (RM) is one way to monitor and manage patients with chronic diseases, without requiring frequent face-to-face appointments. RM for HFrEF is an area of active research, but the majority of studies focus on clinical outcomes such as medication optimization [7], health care usage and mortality [8], rather than user experience [9]. Although user feedback and preferences for smartphone-based RM in cardiovascular disease have been reported in the literature [10,11], it is heavily skewed in favor of patients’ and caregivers’ user experience rather than the nurses’ user experience. Specifically, the impact of RM technologies on nurse user experience and workload is underreported in the literature [12,13]. Furthermore, even in the minority of studies evaluating the user experience of caregivers, the patient perspective is often not simultaneously reported [10]. Therefore, there is a gap in the literature for more studies conducting a balanced evaluation of user experience from both patient and nursing perspectives, using the same type of RM strategy for HFrEF at the same time. A few studies have had this type of design previously, but the RM strategy has been invasive [14] or telephone-based [15], rather than noninvasive smartphone-based RM with patient education. Increasingly, smartphone-based RM is available for HFrEF [16], but the impact of this type of RM on the experience of both patient users and nurse users remains unknown.

In this study, we evaluate the impact of a novel smartphone-based RM platform called Luscii. This strategy of RM combines noninvasive self-measurement of blood pressure, pulse rate and body mass, self-reporting of heart failure, depression and anxiety symptoms, pill usage, a messaging functionality for patient and HFSN communication, and a suite of tailored e-learning modules in a single smartphone app. Specifically, we evaluate usage type and user experience from the point of view of both patients and HFSN users. Additionally, we analyze the change in self-reported vital signs measurements submitted by patients over a 3-month period. Our primary aims are to (1) evaluate how patients and nurses use this type of RM (usage type), (2) evaluate patients’ and nurses’ user feedback on this type of RM (user experience) and (3) directly compare the usage type and user experience of patients and nurses using the same type of RM platform at the same time.

Furthermore, nonadherence to guideline-directed medical therapy for HFrEF contributes to worse clinical outcomes; RM using mHealth strategies may be one way to improve medication adherence [17]. Previous studies have shown a beneficial impact of RM on medication adherence [18], which is usually measured by patients’ self-reported compliance. Guideline-directed medical therapies for HFrEF (such as angiotensin-converting enzyme inhibitors and β-blockers) are known to lower blood pressure and heart rate, whereas body mass is often used as a measure of effectiveness and adherence to loop diuretics [19]. In this study, we additionally hypothesize whether RM of vital signs such as blood pressure, pulse rate, and body mass could be a useful surrogate for medication adherence that does not depend on patients reporting for themselves whether they have taken their tablets. Therefore, additionally, our secondary aim is to investigate whether there is a significant change in blood pressure, heart rate, and body mass in the first 3 months of using this type of RM platform.

## Methods

### Study Design

Using a mixed methods approach consisting of qualitative free-text thematic analysis and quantitative analyses of vital signs measurements, we retrospectively analyzed the usage type and user experience of two groups of users of the RM platform:

1. Patient users: patients with HFrEF (index left ventricular ejection fraction [LVEF] <40% measured by echocardiography) being treated in our regional heart failure service in London, United Kingdom. The inclusion criteria were consenting to using and being onboarded to the RM platform between April 2021 and November 2021 and having submitted at least 2 measurements per week for at least 3 months. Demographic data, medical comorbidities, and heart failure severity (measured by LVEF on echocardiogram and New York Heart Association class) were extracted from the electronic health record.
2. HFSN users: the cohort of HFSNs at our hospital who routinely used the RM platform to manage patients with chronic HFrEF.

### Ethics Approval

Institutional approval was granted by the Imperial College Healthcare National Health Service (NHS) Trust Audit and Quality Improvement Committee (Ref CAR/077). Participants were informed that their feedback would be used anonymously for audit and research. All participants consented to the use of their anonymous responses by participating in the feedback or focus group. Patients used their own mobile devices to run the RM app.

### Data Collection

For the patient user group, all users were invited to submit free-text comments via the RM platform. There were no specific questions asked, but patients were told that they could use the unstructured free-text response field to express their feedback about topics such as the RM platform itself, their reasons for using RM, how they used it, their positive and negative experiences of RM, and their views on RM in general. Text comments were extracted from the RM database by bespoke searches written in the SQL query language.

Self-measured vital signs (pulse rate, blood pressure, and body mass) submitted by patients to the RM platform were also extracted at two timepoints: (1) at onboarding (week 1 of platform use) and (2) 3 months after onboarding (week 12 of platform use). To be included in this part of the analyses, patients had to have submitted at least 5 measurements in the first week and in the 12th week of platform use (ie, at both timepoints). To enable robust comparison of average readings, rather than analyzing single values of measurements that have high interday and intraday variability, the mean value of all measurements submitted in week 1 and week 12 was calculated. The difference in mean systolic blood pressure, diastolic blood pressure, heart rate, and body mass between week 1 and week 12 was compared.

For the HFSN user group, we conducted a focus group of HFSNs who managed patients with HFrEF using the RM platform. The focus group was semistructured, allowing HFSNs to express their feedback on a range of issues pertaining to the RM platform. The topics discussed were as follows:

1. How HFSNs used the RM platform
2. Their perceived positive impacts of this type of RM
3. Their perceived negative impacts of this type of RM
4. Their views on smartphone-based RM for HFrEF in general

The focus group was facilitated by a trained member of the research team and transcribed. The text comments from patient users and HFSN users were combined with the HFSN focus group transcription, resulting in a single large text data set of user feedback for qualitative analyses (Multimedia Appendix 1).

### Primary Analyses: Semantic Analysis of Usage Type and User Experience

The text data generated from the patient user feedback and the HFSN focus group were thematically analyzed using the method described by Braun and Clarke [20] by 2 independent members of the research team. The following stages of analysis were used: familiarization with the data, generating initial codes, searching for themes or subthemes, reviewing themes or subthemes, defining and naming themes or subthemes, producing the final report, and checking validity.

Detailed methodology for these analyses is described in Multimedia Appendix 1. The final themes, subthemes, and relevant quotation examples from the raw data were identified for presentation in the results.

### Secondary Analyses: Difference in Vital Signs Over 3 Months of Platform Use

These analyses pertained only to the self-measured vital signs data collected from patients after week 1 and week 12 of RM platform use.

Paired 2-tailed *t* tests were used to evaluate the difference between mean systolic blood pressure, diastolic blood pressure, heart rate, and body mass between week 1 and week 12. For each type of measurement, the null hypothesis was that there was no statistically significant difference between the mean measurement at onboarding and the mean measurement after 3 months. *P* values of <.05 were deemed statistically significant.

### The RM Platform

The RM intervention in this study used the Luscii platform. This is a commercially available smartphone-based RM platform.

The intervention combined three modules within a single smartphone app:

1. Measurements module (Figure S1 in Multimedia Appendix 2): patients were given a digital sphygmomanometer, pulse rate monitor, and body mass scale connected to the smartphone app via Bluetooth. Patients were prompted to submit measurements daily, with no upper limit on the number of allowable measurements. All previously submitted measurements were viewable by the patient and clinicians in graphical and tabulated formats. Patients could also complete optional questionnaires about heart failure symptoms, pill usage, anxiety, and depression.
2. Self-care module (Figure S2 in Multimedia Appendix 2): e-learning modules written by HFSNs in our department were uploaded to the Luscii app. These covered topics such as prognostic heart failure medication, information about different cardiac investigations, and device therapy.
3. Messages module (Figure S3 in Multimedia Appendix 2): patients had the option to add free-text comments to their measurements, which were sent to clinicians in the form of messages. In this module, clinicians (typically HFSNs) could respond to these messages or send new messages as unstructured free text. HFSNs were available to interact with patients using this module between 9 AM and 5 PM, Monday to Friday.

Screenshots of the different modules in the Luscii platform are shown in Multimedia Appendix 2.

From the clinician-facing side of the platform, HFSNs could review previous measurements of vital signs, review responses to the Heart Failure Questionnaire and anxiety and depression questionnaires, view comments and messages sent by patient users, send messages to patient users, set personalized thresholds for vital signs to automatically alert HFSNs, and upload heart failure educational material through e-learning modules.

## Results

### Participant Characteristics

A total of 83 patients with HFrEF were onboarded onto the RM platform between April 2021 and November 2021; 4 patients used the platform for fewer than 3 months (2 dropped out and 2 died), so 79 patients (mean age 62 years; 35%, 28/79 female) were included in the analyses. Demographic data, medical comorbidities, and heart failure severity of the patient users included are shown in Table 1.

**Table 1:** Characteristics of 79 patient users with heart failure with reduced ejection fraction who were onboarded to the remote monitoring platform and used it for at least 3 months.

Characteristic at the point of onboarding to the remote monitoring platform		Value
**Demographics**		
	Age (years), mean (SD)	62.0 (13.4)
	Female, n (%)	28 (35)
**Ethnicity, n (%)**		
	White	43 (55)
	Black	13 (16)
	Asian	8 (10)
	Mixed	7 (9)
	Other	8 (10)
**Medical comorbidities, n (%)**		
	Ischemic heart disease	24 (30)
	Atrial fibrillation	22 (28)
	Hypertension	27 (34)
	Stroke	5 (6)
	Type 2 diabetes mellitus	13 (16)
	Chronic obstructive pulmonary disease	10 (13)
	Chronic kidney disease	8 (10)
**Heart failure parameters**		
	Left ventricular ejection fraction (%), mean (SD)	32 (11)
**New York Heart Association classification, n (%)**		
	I	12 (15)
	II	39 (49)
	III	23 (29)
	IV	5 (7)

### Primary Results: Usage Type and User Experience

Of the 79 patients, 58 (73%) submitted feedback in the form of text comments via the RM platform. A total of 6 of 9 (67%) HFSNs participated in the focus group.

#### Usage Type

**Figure 1:**
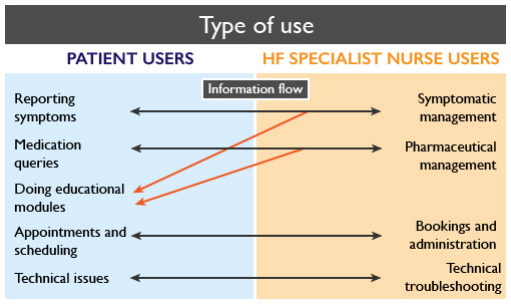
Different ways the patient users and heart failure specialist nurse (HFSN) users used the remote monitoring platform. The arrows indicate the direction of information flow.

The RM platform enabled bilateral information exchange between patients and HFSNs. Both user groups used the platform to exchange information on a wide range of topics including reporting symptoms, medication queries, appointments, and administration and technical issues (Figure 1). HFSNs were able to create e-learning modules that were delivered via the RM platform. These educational modules were another major method of information exchange between the 2 user groups and aided delivery of advice regarding symptoms and medications.

#### User Experience

Thematic analysis of free-text questionnaire responses and focus group data revealed 2 key themes for each user group: positive impacts and negative impacts. Within these themes, there were multiple subthemes that overlapped between the patient users and HFSN users (Table 2).

**Table 2:** Thematic analysis of free-text comments from 58 patient users and a focus group of 6 heart failure specialist nurse users of the remote monitoring platform. Two main themes are of positive and negative impacts. Different subthemes for each user group are nested within each theme. Examples of text data within each subtheme are provided as quotations.

Theme and Subtheme (examples)				
Patient users		Heart failure specialist nurse users		
**Positive impacts**				
	Increased engagement and understanding: (“...makes me pay closer attention to my weight and blood pressure.”) Reassurance and security: (“I feel much safer...peace of mind knowing there is a team watching over me.”) More convenient: (“...makes me feel...protected without the inconvenience of being in hospital”; “...I don’t have to rely on nurses coming round to do blood pressure checks.”) Early abnormality detection: (“...makes it possible to take actions in advance to prevent heart attacks.”) Enhanced communication: (“...allows patients to express their concerns and knowing there is somebody there who will listen and reply to them.”)	Increased engagement and understanding: (“...promotes patients being more proactive in self-management of their condition.”) Enhanced usual care: (“...good adjunct to usual care, does not replace but enhances.”) Admissions avoidance: (“...we have avoided admissions”; “...useful way to...prevent hospital admissions.”) Early abnormality detection: (“...allows trends to be spotted more quickly and actions to be taken for patients deteriorating or at risk of hospital admissions.”) Medication optimizations: (“...useful aid when titrating medications remotely.”)		
**Negative impacts**				
	Lack of human interaction: (“...having a ‘human’ voice to talk to is far better.”) Information overload: (“I check it too often and read too much or too little into it.”) Technical issues: (“...when I can’t get it to connect it gets me very frustrated...”)	Increased workload: (“Sometimes can be difficult to manage the additional alerts”; “On-boarding can be complicated and time-consuming for staff.”) Accessibility limitations: (“...only suitable for those that are tech savy and access to a smartphone.”) Technical issues: (“Very much dependent on whether connections are good.”)		

Overall, the positive subthemes outnumbered negative subthemes. Both user groups reported that the RM enabled early detection of abnormalities while increasing patient engagement and understanding of HFrEF. Patient users felt that RM was more convenient than traveling to attend face-to-face appointments, offered them reassurance, and enhanced communication with HFSNs. HFSN users reported efficiency gains due to fewer appointments, admission avoidance, and medication optimization.

Negative subthemes from the analysis of patient user feedback included information overload and a lack of human interaction compared with face-to-face appointments. Analysis of negative HFSN user feedback revealed concerns that RM monitoring was not accessible to all and highlighted the potential of increased clinical workload. The negative subtheme of technical issues was symmetrically reported by both user groups (Table 2).

### Secondary Results: Impact of RM on Self-measured Vital Signs

The majority of patients provided measurements of pulse rate (73/73, 100%), blood pressure (70/73, 96%), and body mass (65/73, 89%) at both timepoints. Missing data were handled using complete case analysis (only patients who had sufficient measurements to calculate an average at both timepoints were included). For the patients who had incomplete data, no obvious relationship was found between them at either variable level or timepoint level. The missing data were therefore deemed to be missing completely at random and unlikely to bias the complete case analysis approach.

Post hoc calculations found that at least 64 patients were required to detect a 10% decrease in blood pressure, heart rate, and body mass between the 2 timepoints at an α of .05 and power of 80%. On average, after 3 months of RM use, there were significant reductions in pulse rate (*P*=.004) and blood pressure (systolic *P*=.008*;* diastolic *P*=.002) but no change in body mass (*P*=.97; Table 3).

**Table 3:** Table3. Self-measured vital signs for patients using the remote monitoring platform. Values were measured at 2 timepoints: at onboarding to the platform and 3 months later.

Patient-measured parameters	Onboarding, mean (SD)	After 3 months, mean (SD)	*P* value
Heart rate (bpm; n=79)	73 (13)	69 (10)	.004
Systolic blood pressure (mm Hg; n=76)	123 (19)	119 (16)	.008
Diastolic blood pressure (mm Hg; n=76)	76 (12)	73 (10)	.002
Body mass (kg; n=70)	86.4 (24.2)	86.4 (22.6)	.97

## Discussion

### Principal Findings

In this study, we present a balanced evaluation of how a smartphone-based RM platform is used by patients with HFrEF and HFSNs at the same time and their respective user experiences over a 3-month period. We also report the change in vital signs after 3 months of RM use. This study has 4 key findings. First, this type of RM is feasible in this population (dropout rate 2% over 3 months). Second, the RM platform was used for sharing bilateral information between patients and HFSNs. Third, both user groups reported predominantly positive impacts on their experience, and there was considerable overlap in the type of experience reported by each group. Finally, after 3 months of RM platform use, there was a significant reduction in blood pressure and heart rate, but not body mass.

### Qualitative Benefits of Smartphone-Based RM

The feasibility of RM in patients with HFrEF observed in this study is in line with previous work [9]. Although the majority of research in this area is to do with the impact of RM on clinical outcomes [6], comparisons between patient and nurse user experience for the same type of RM at the same time are less well known [21]. This study fills this gap in the literature by providing a balanced analysis of the qualitative impact on both user groups.

Both user groups reported that this type of RM was more convenient than serial face-to-face appointments. This is particularly relevant for heart failure because patients with HFrEF have high rates of frailty and low mobility, which is independent of age [22]. From a nurse perspective, smartphone-based RM is likely to be more convenient than telephone-based RM because measurements and communication can be conducted asynchronously [23]. As previously described, this gives HFSNs more flexibility to fit the RM tasks around other clinical commitments [16].

The patient users in this study expressed that RM had a positive impact on continuity of care, engagement, awareness, and feelings of safety. This was mirrored by HFSNs who reported that their interactions with patients via the RM were more fulfilling. This is in line with previous work [14,24]. This type of RM may improve continuity of care, which is particularly important to patients with HFrEF. Our study supports previous findings that improving bilateral continuity of care may increase the adoption and engagement with RM technology for both user groups [25].

Our analyses also revealed some unexpected uses of the RM platform. This included symptom reporting and appointment scheduling (Figure 1). Although this was not an intended purpose of the platform, it illustrates that users are able to creatively adapt their use to maximize functionality and convenience. In this way, smartphone-based RM may have additional utility beyond just clinical optimization. Indeed, patients taking an active role in the timing and frequency of their follow-up may be a measure of increased “self-management” (when patients monitor their own signs and symptoms, adhere to treatment, are able to recognize changes in their clinical state, and respond to these by altering their behavior or seeking assistance).

### Impact on Medication Adherence

Improving medication adherence is a key aim of RM in HFrEF [26]. In this study, we considered reduction in heart rate, blood pressure, and body mass as a possible surrogate for investigating medication adherence in our secondary analyses. Previous studies have shown that eHealth self-management interventions such as RM can improve medication adherence in heart failure [18]. Compared with when they were onboarded, we found that patients had significantly lower blood pressure and heart rate after 3 months of RM use. This may reflect adherence with prognostic medications such as angiotensin-converting enzyme inhibitors, β-blockers, and mineralocorticoid receptor antagonists. Interestingly, there was no significant difference in body mass, a metric that is often used to assess the overall fluid status and degree of fluid overload and to guide titration of diuretics. The lack of reduction in body mass in this study may be because patients were not too fluid overloaded at onboarding (almost 50% of patients were only New York Heart Association II at the point of starting RM, ie, they had mild symptoms); therefore, they had relatively little fluid to lose in the first place.

### Potential Downsides of RM

Our analyses revealed that the user perceptions of smartphone-based RM for HFrEF were not universally positive. First, HFSNs reported an increased workload due to checking and responding to alerts on the RM platform, which was typically in addition to existing clinical commitments. This is in line with previous studies that have reported greater nursing activity for patients having telehealth monitoring; in one study, nurses had twice as much activity with RM patients as with controls [27]. However, almost a third of the activities were to do with the provision of health information or lifestyle education. The RM strategy in our study has built-in e-learning for self-care and education. Aside from being more convenient for patient users, compared with telephone-based monitoring, this may be an important intervention to enable users to benefit from the upsides of RM without overburdening HFSNs with the responsibility of providing synchronous patient education. As RM becomes increasingly prevalent in clinical practice, we recommend that organizational routines and reimbursement be adjusted to specifically account for this additional activity [14]. Furthermore, developers of RM platforms should be mindful not to overwhelm the nurse users with excessive alerts that are known to be distracting [28]. This is in line with a previous study that found that RM caused some nurse user distress due to increased responsibility and workload [14]. We support their recommendations to adjust organizational routines and reimbursement systems to give nurse users more security when using RM technology.

Second, patient users experienced some information overload. This potential pitfall has been reported previously for another type of monitoring technology [29] and can also affect clinicians [30]. The risk of information overload may indeed be higher for this smartphone-based RM than telephone-based RM because the ease and convenience (any time of the day, with no capping of the number of measurements allowed) of the former is likely to generate much more data than traditional RM approaches. We recommend that developers be mindful not to create platforms that overwhelm users, leading to lower usability and more inefficiency.

Third, both user groups reported technical issues with the RM platform. This led to frustration from patient users and inefficiency for HFSNs. Although the inevitability of some technical issues is appreciated by previous work [31,32], there is a dearth of studies evaluating their impact on the user experience. This study highlights that these issues can have substantial negative impact on user experience for both patients and nurses. Technical issues risk undermining trust in the RM platform, which may have implications for wider adoption and acceptance. This also highlights the importance of having end users involved in the development and testing stage of smart RM technologies [33]. Further research should be directed to evaluating the extent and impact of technical issues on the quality of user experience.

### Impact of Smartphone-Based RM on Health Inequalities

The risk of RM technologies increasing health inequalities was a negative subtheme reported by HFSN users. Nurses expressed concern that this type of technology risked excluding patients who did not own smartphones or were not technologically savvy. Socioeconomic status is one driver of RM adoption [34]. This is supported by the fact that our cohort of patient users were on average from a higher socioeconomic class (measured by the indices of multiple deprivation [35]) than the general population (median indices of multiple deprivation decile 3 vs 5).

Age is another important factor. The mean age for a new heart failure diagnosis in the United Kingdom is 76.6 years [36]. The mean age of the cohort of patient users in this study is much less (62 years). This reflects the fact that older patients, in general, did not opt for this RM strategy, which is in line with previous research [37]. As a result, we recommend that RM should be viewed as a supplement to, not a replacement for, usual guideline-directed clinical care. Smartphone RM may optimize management remotely for those who choose it, enabling redistribution of resources to enhance standard care for those who are unwilling or unable to have RM [34]. With smartphone use becoming ever more prevalent [38], the proportion of patients unable to use smartphone RM technology will also reduce [39]. We recommend that health care providers be mindful of the risk that RM technology could increase rather than reduce health inequalities and concerted efforts to engage a broad user group while maintaining a high quality of usual care so that those to choose not to have RM are not worse off [40].

### Limitations

First, this study evaluates the RM experience of patients and HFSNs at one center in one part of London. More studies are needed with a larger sample size to replicate these findings before practice recommendations can be made.

Second, not all eligible participants participated in all analyses; however, the response rate was still high: the response rate for patient feedback was 73% (53/73), 67% (49/73) of HFSNs participated in the focus group, and >89% of patients were included in vital signs analyses. Furthermore, patients were not obligated to provide any feedback, so there may be a selection bias of opinions skewed in favor of those who chose to. However, we did not find any relationship that linked the patients for whom data were missing. Therefore, it is unlikely that the missingness of these data substantially biased our analyses. Nevertheless, the majority of eligible participants contributed data to each analysis so the results can be seen to be widely representative of the population studied. Future studies should aim to increase the response rate further so that the full gamut of user opinion is captured and use alternative methods of handling missing data such as multiple imputation if the reason for missing data turns out to be nonrandom.

Third, in this study, we evaluated the initial impact of this technology on its users over a 3-month period. This was our experience in the first 3 months using this type of RM in our region. Previous studies have shown that adherence to RM itself reduces with time [26]. Further research to see whether the impact we found in this study is sustained in the long term is ongoing.

Fourth, during the study period, the RM platform was licensed only for use in patients with HFrEF (LVEF <40%). We have not analyzed how it impacts patients with preserved ejection fraction. These patients make up a large proportion of the heart failure population, and future research should include their experiences and comparison of these experiences with those of patients with HFrEF.

Finally, our secondary findings relating to the use of vital signs as a surrogate for medication adherence should be contextualized within the limitations of possible biases of self-measurement, inter- and intraday variation, the lack of a non-RM comparator arm, and the fact that there are no corresponding prescription data in this study. However, it may suggest a way for future studies to leverage RM of vital signs to measure adherence to medical therapy.

### Conclusions

Smartphone-based RM of vital signs with integrated bilateral information sharing and patient education is feasible in HFrEF. Over a 3-month period, this platform had positive impacts on patient users such as increased convenience, reassurance, and self-care. A significant reduction in blood pressure and heart rate over 3 months may reflect good adherence to guideline-directed medical therapy and warrants further investigation. Nurse users reported symmetrical impacts including more continuity and the potential for admission avoidance. We found potential pitfalls, such as information overload for patients, increased workload for nurses, and technical issues for both user groups. To maximize RM adoption and acceptance, we recommend that RM providers actively involve both patient and nurse users in platform development and that managers formally recognize time spent using RM in nursing job plans.

## Appendix

Multimedia Appendix 1 Detailed methods for thematic (semantic) analysis of text data from patient user feedback and a heart failure specialist nurse user focus group.

Multimedia Appendix 2 Screenshots of the remote monitoring intervention smartphone app.

## Abbreviations

HFrEF: heart failure with reduced ejection fraction
HFSN: heart failure specialist nurse
LVEF: left ventricular ejection fraction
NHS: National Health Service
RM: remote monitoring
