# Supplementary material for: Smartphone-Based Remote Monitoring for Chronic Heart Failure: Mixed Methods Analysis of User Experience From Patient and Nurse Perspectives
Source: JMIR Nurs. 2023 Jun 6;6:e44630. doi: 10.2196/44630 (PMC10282903; doi:10.2196/44630)
Supplement: Multimedia Appendix 1 [file nursing_v6i1e44630_app1.docx]

**Multimedia Appendix 1: Detailed methods for thematic (semantic) analysis of text data from patient user feedback and HFSN user focus group**

The participants in these analyses were:

1. The patient users of the RM platform who provided written feedback (unstructured text) via the RM platform itself. Their responses were collated into a single document after querying the RM SQL database.
2. The HFSN users who participated in the focus group discussions The focus group was held virtually via video conferencing lasting 1 hour, recorded and transcribed.

Together this generated a single dataset of combined feedback from patients and HFSNs. This combined dataset was analyzed for the purpose of this study.

**Thematic Analysis**

Thematic analysis was conducted using the method proposed by Braun and Clarke (2006)[20]. The analysis consisted of six stages:

Stage 1: Familiarization with the Data

The transcribed text data were read and re-read several times by two researchers working independently (i.e. blinded to each other’s’ analyses) to gain a thorough understanding of the data. Notes were taken on initial thoughts and observations. The only guidance that the researchers were given was to consider the overall study aims of evaluating the ‘usage type’ and ‘user experience’.

Stage 2: Generating Initial Codes

Next, the data were coded inductively by hand. Each element of data was read through line by line, and relevant sections were highlighted and labeled with codes. Codes were generated based on the content of the data, rather than predetermined categories or themes. The initial codes were recorded in a spreadsheet for further analysis.

Stage 3: Searching for Themes

After the initial codes were generated, we reviewed the codes and sorted them into potential themes. Similar codes were grouped together, and potential themes were identified based on the shared content of the codes. At this stage, we discussed and refined the themes until a final list of potential themes was created.

Stage 4: Reviewing Themes

The potential themes were reviewed and defined in more detail. This involved checking the codes under each theme and ensuring that they fit together cohesively. The themes were named and described in more detail, and a codebook was created to define each theme and its associated codes.

Stage 5: Defining and Naming Themes

Once the final themes were agreed upon, we reviewed the transcripts again to ensure that each theme was well-supported by the data. This involved checking that each code fit into the correct theme and that no codes were left out. Any necessary revisions were made to the codebook at this stage.

Stage 6: Producing the Final Report

The final stage involved writing up the results and producing a report that described the themes, sub-themes, and codes that emerged from the analysis. The report included quotes from the raw text data to illustrate each theme and subtheme. We checked the report for accuracy and coherence before finalizing it. This report was presented at a consensus meeting of researchers.

Validity

To ensure the validity of the analysis, we used several strategies. First, they checked for inter-rater reliability by having two researchers independently code a subset of the data and comparing their codes. Any discrepancies were discussed and resolved at the consensus meeting, with a third member of the research team arbitrating over any cases of non-agreement. We ensured that the analysis was transparent by providing a detailed description of the analytical process and including quotes from the data to illustrate each theme in our results section.
