# Supplementary material for: Smartphone-Based Remote Monitoring for Chronic Heart Failure: Mixed Methods Analysis of User Experience From Patient and Nurse Perspectives
Source: JMIR Nurs. 2023 Jun 6;6:e44630. doi: 10.2196/44630 (PMC10282903; doi:10.2196/44630)
Supplement: Multimedia Appendix 2 [file nursing_v6i1e44630_app2.docx]

**Multimedia Appendix 2: Screenshots of the remote monitoring intervention smartphone app**


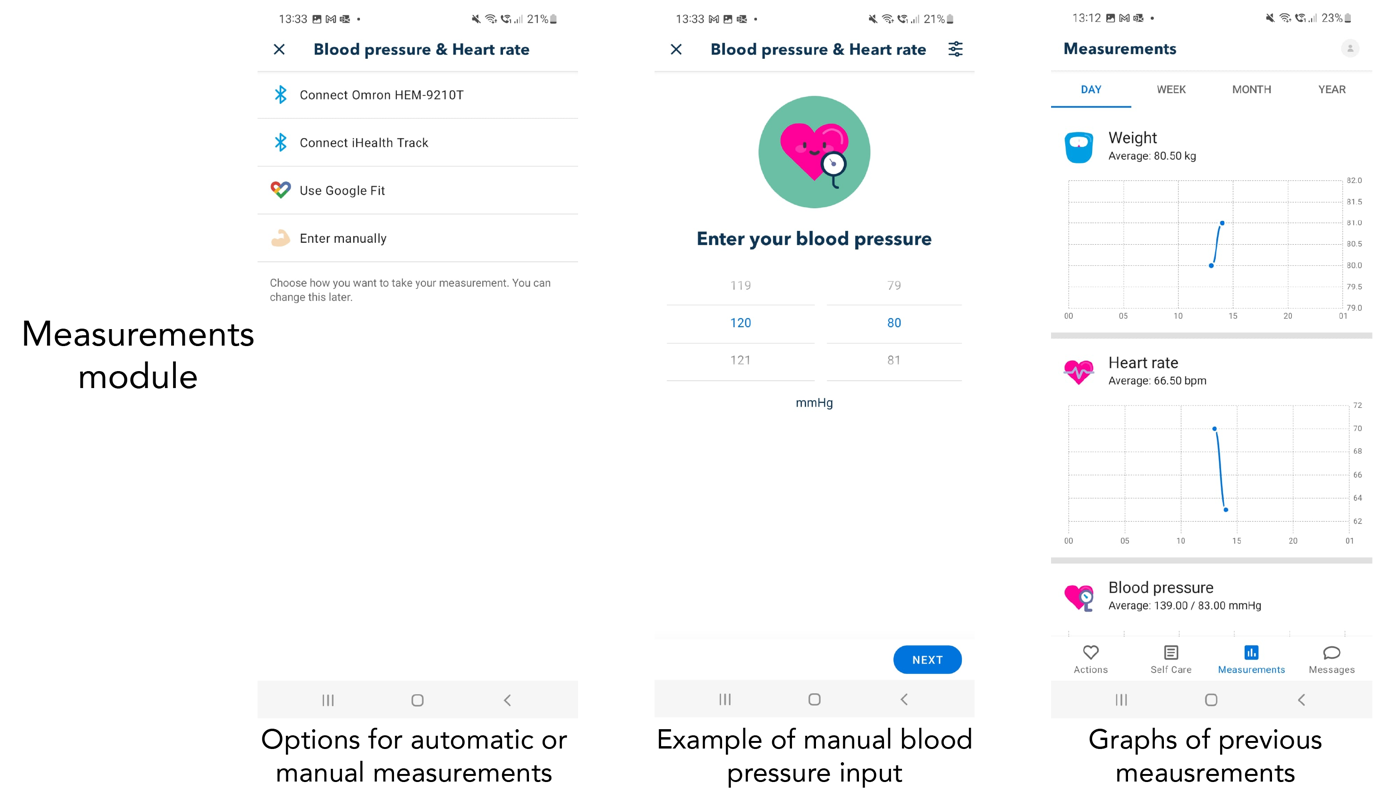


Figure S1: Screenshots of the measurements module in the Luscii smartphone app.

**
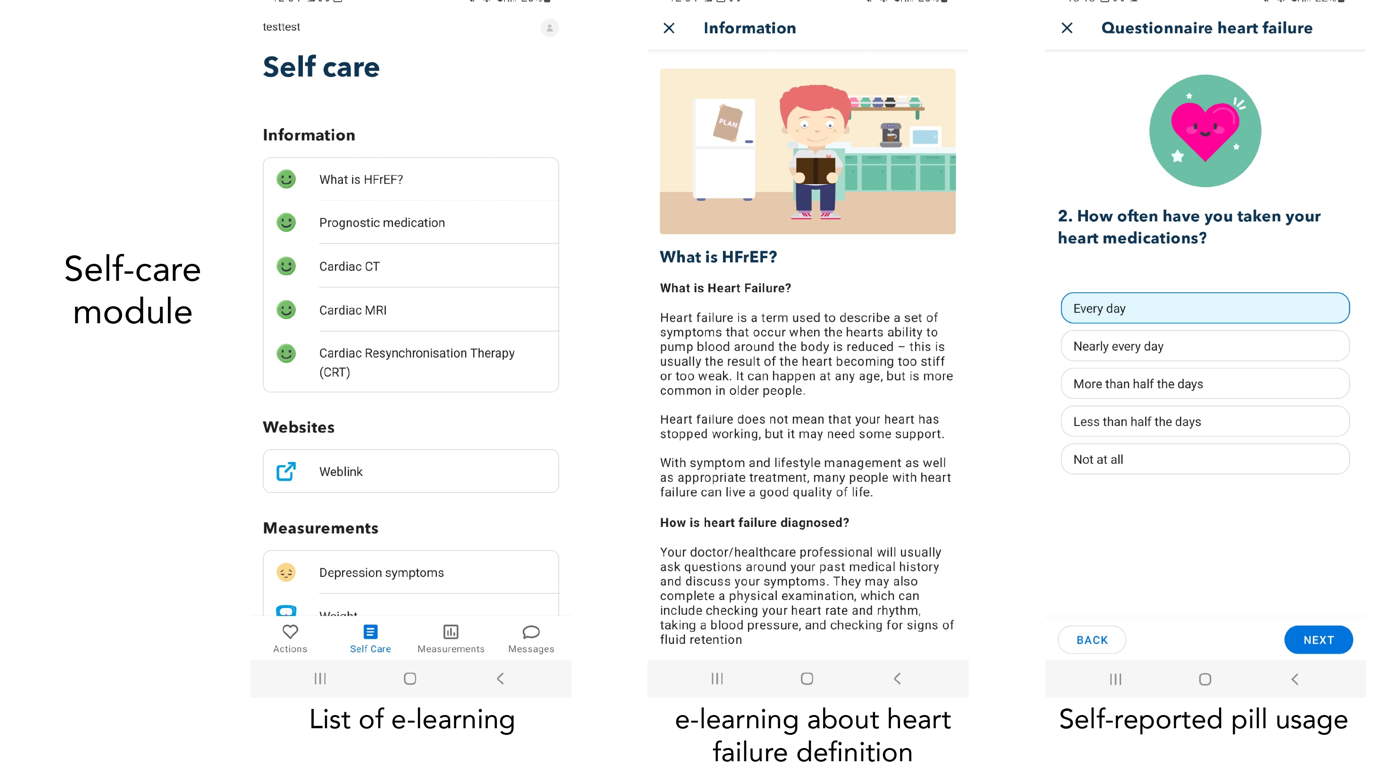
**

Figure S2: Screenshots of the self-care module in the Luscii smartphone app.

**
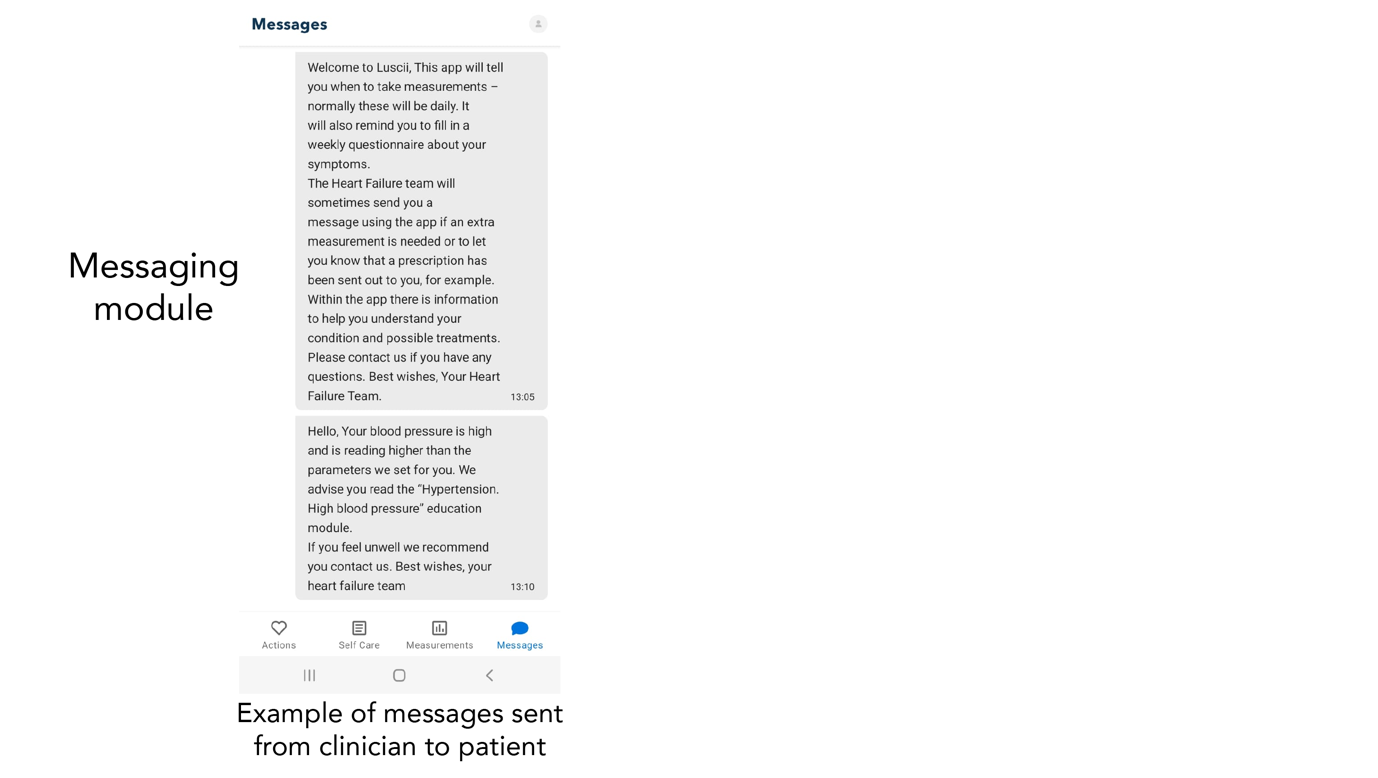
**

Figure S3: Screenshot of the messages module in the Luscii smartphone app.
